# Supplementary figures and images for: Assessing quantitative MRI techniques using multimodal comparisons
Source: PLoS One. 2025 Jul 24;20(7):e0327828. doi: 10.1371/journal.pone.0327828 (PMC12289042; doi:10.1371/journal.pone.0327828)

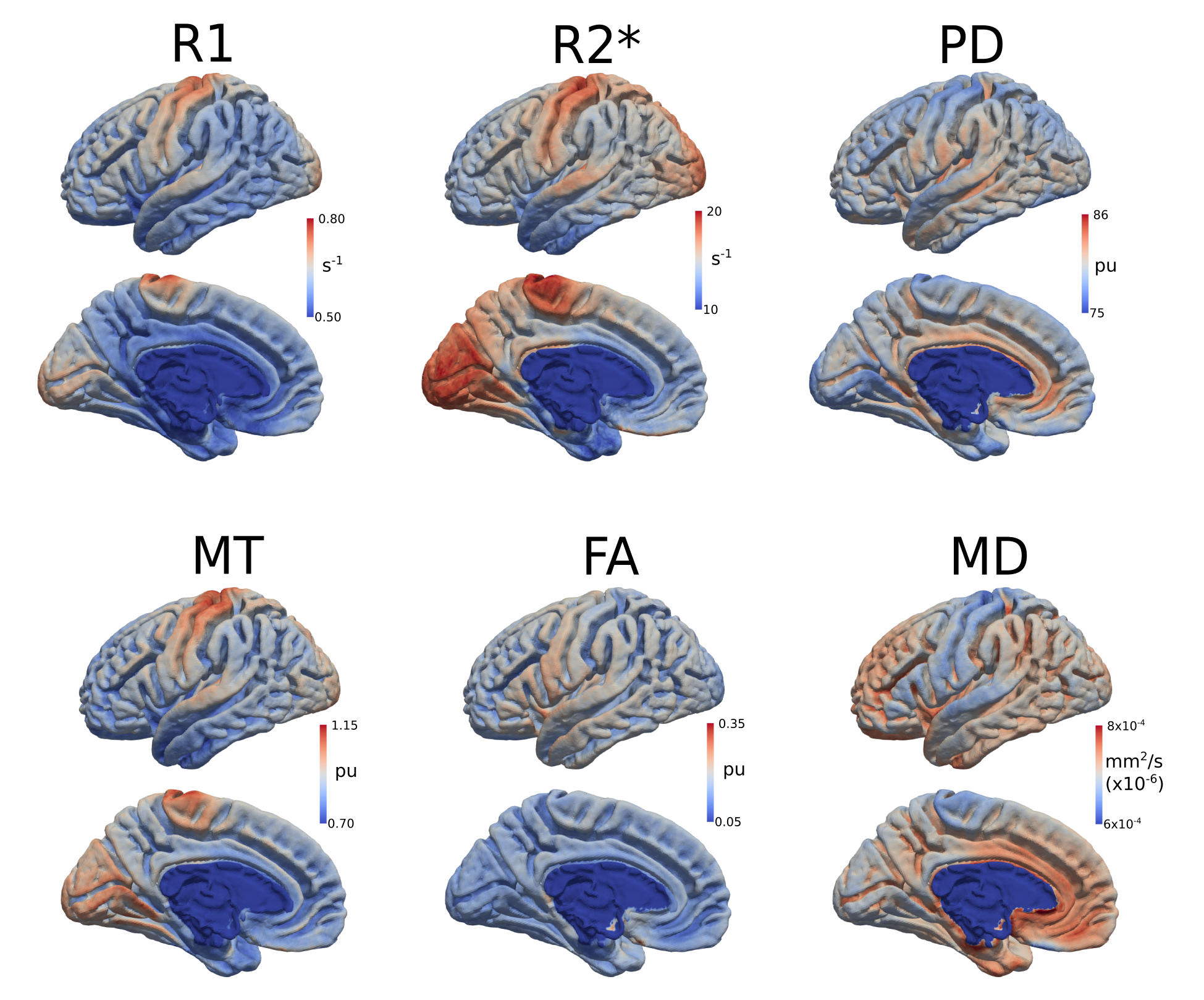

Supplement: S1 Fig — Median values from the cortical ribbon across all participants are plotted on the cortical surface. The range of values are indicated on the colorbar for each metric (pu = percentage units). (TIF) [file pone.0327828.s001.tif]

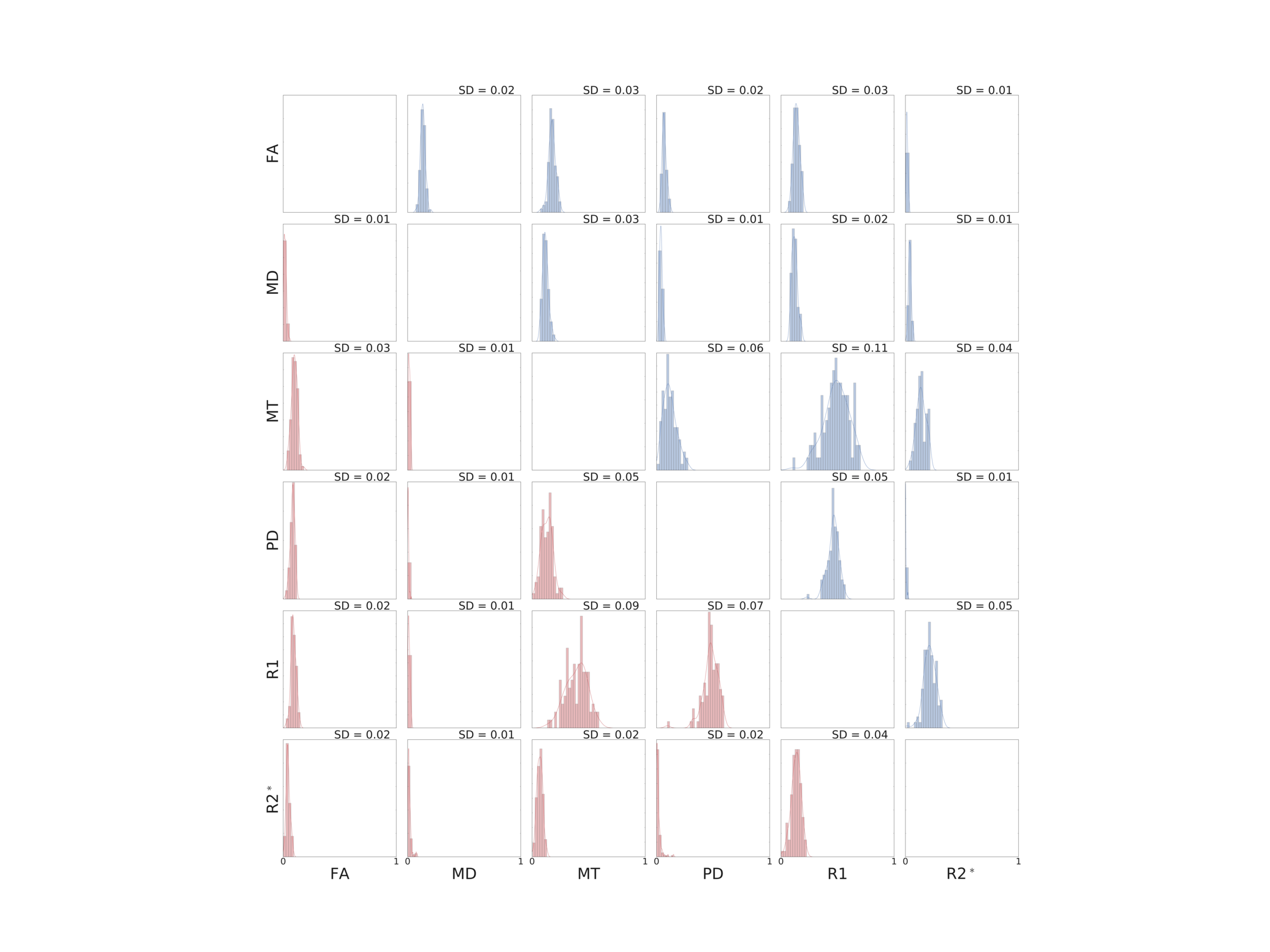

Supplement: S2 Fig — This shows that the relationships between metrics were highly consistent across individuals. (TIF) [file pone.0327828.s002.tif]

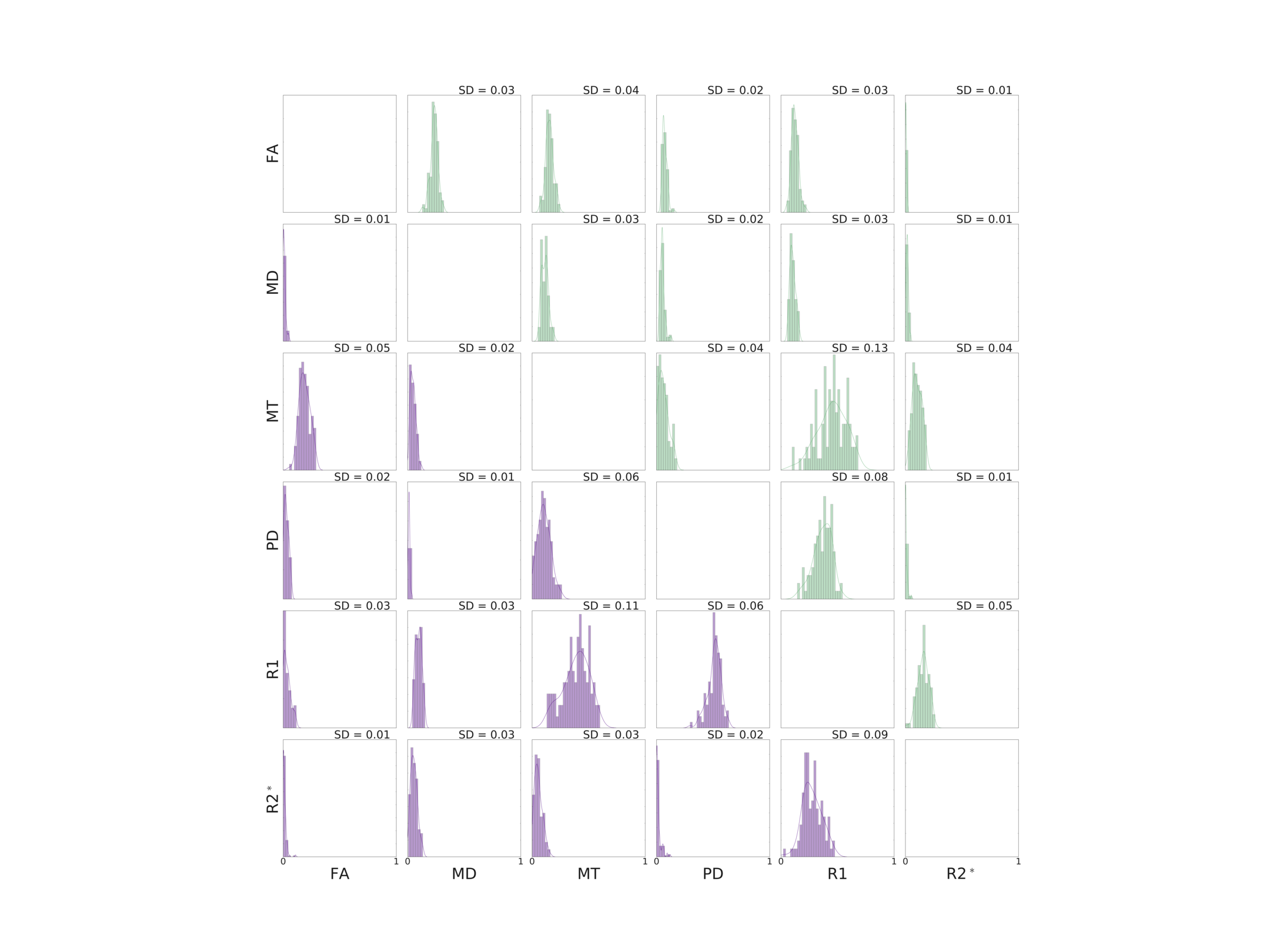

Supplement: S3 Fig — This shows that the relationships between metrics were highly consistent across individuals. (TIF) [file pone.0327828.s003.tif]

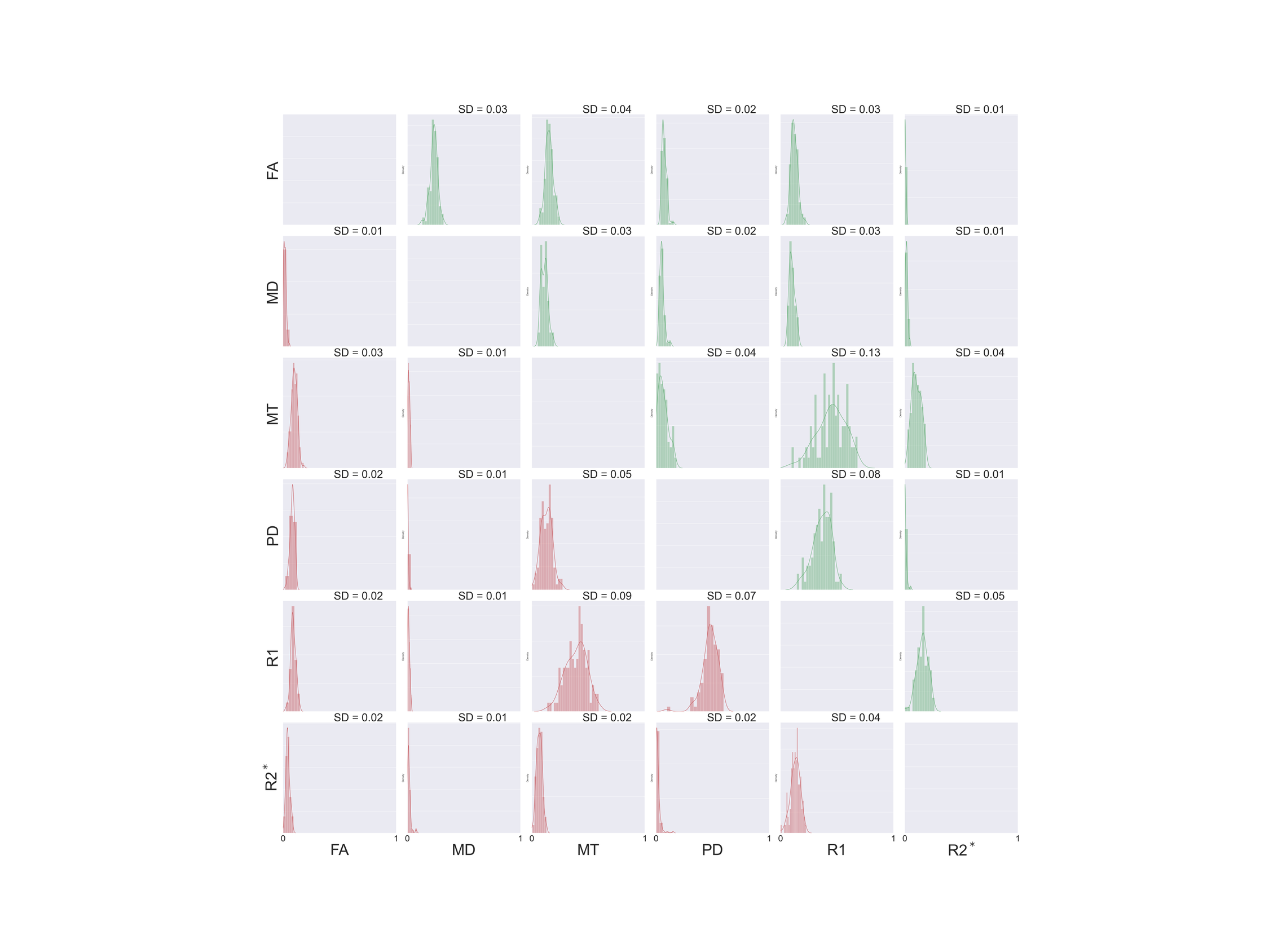

Supplement: S4 Fig — This shows that the relationships between metrics were highly consistent across individuals. (TIF) [file pone.0327828.s004.tif]

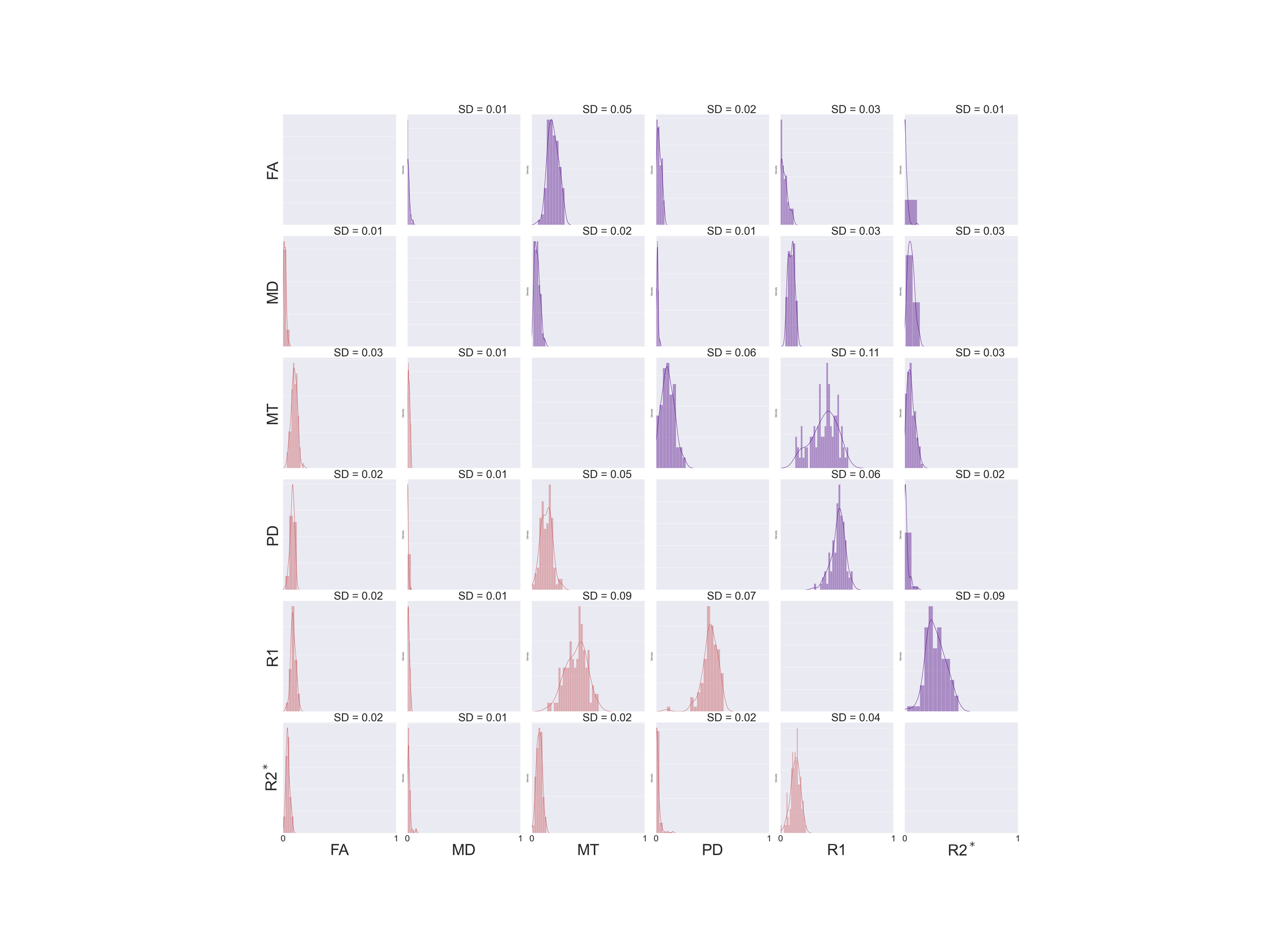

Supplement: S5 Fig — This shows that the relationships between metrics were highly consistent across individuals. (TIF) [file pone.0327828.s005.tif]

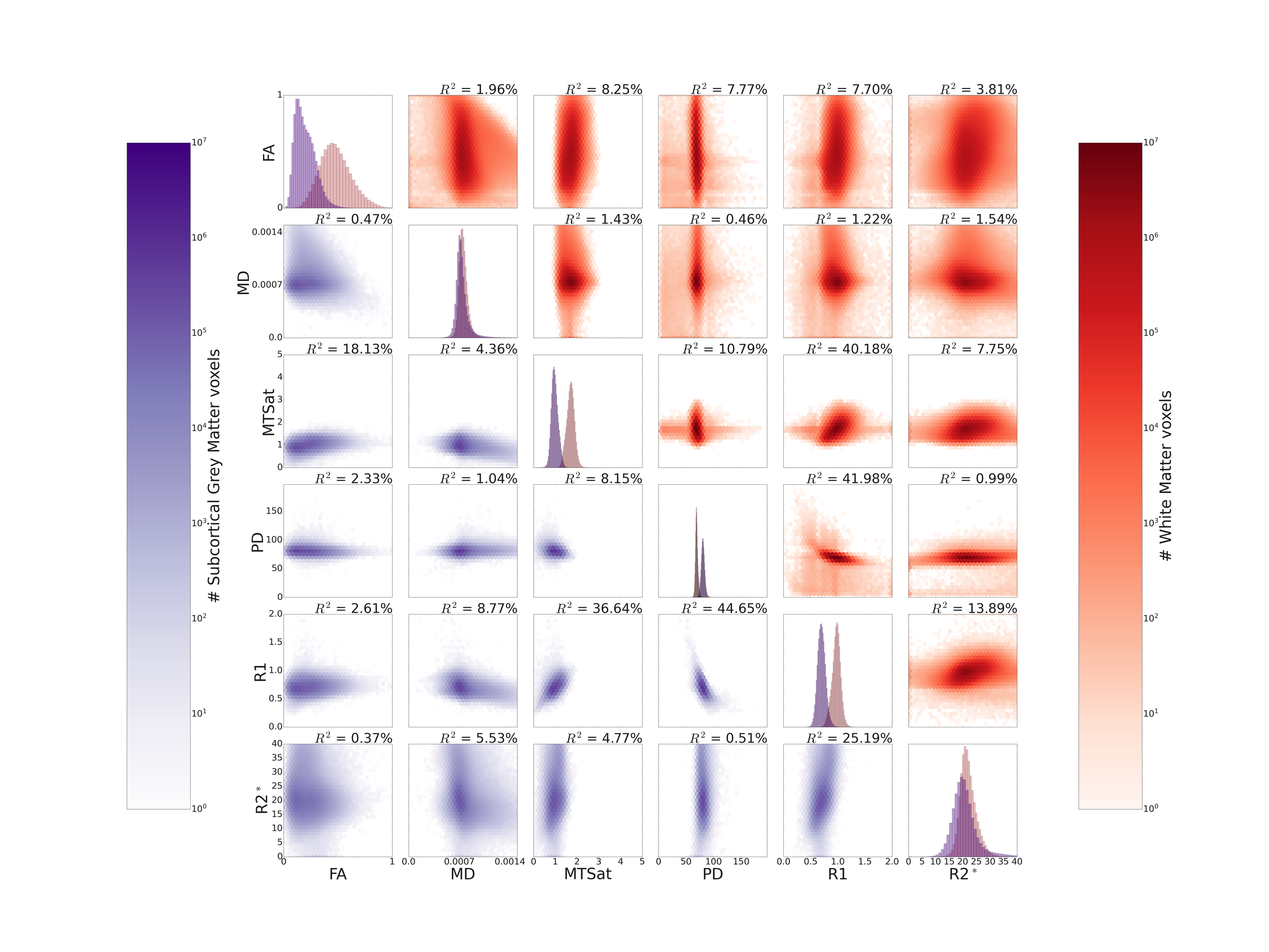

Supplement: S6 Fig — P-values for all pairwise correlations were very small (<0.001), however were not stated explicitly as the small values of p do not necessarily relate to higher explained variance. R2 values for each pairwise correlation are shown above their respective graphs Data along the diagonal shows the histograms for each metric in subcortical (purple) and WM (red). Metric thresholds were implemented for R2* and MD to reduce partial volume effects and artifacts, and are visible as sharp cutoffs in the figure (see Method). Pairwise density plots are depicted in log space to show the full range of data. (TIF) [file pone.0327828.s006.tif]

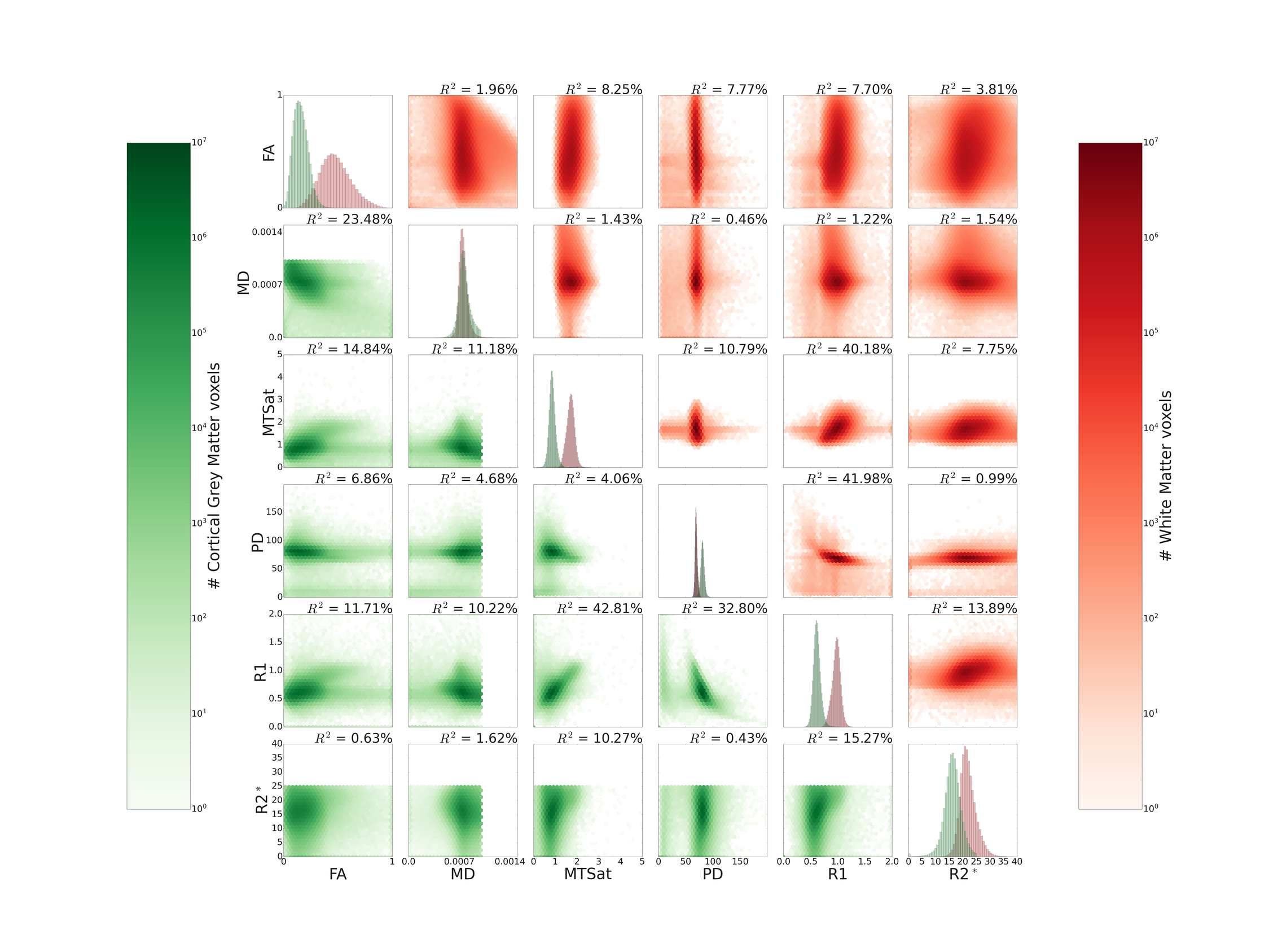

Supplement: S7 Fig — P-values for all pairwise correlations were very small (<0.001), however were not stated explicitly as the small values of p do not necessarily relate to higher explained variance. R2 values for each pairwise correlation are shown above their respective graphs Data along the diagonal shows the histograms for each metric in cortical (green) and WM (red). Metric thresholds were implemented for R2* and MD to reduce partial volume effects and artifacts, and are visible as sharp cutoffs in the figure (see Method). Pairwise density plots are depicted in log space to show the full range of data. (TIF) [file pone.0327828.s007.tif]
