# Supplementary material for: Assessing quantitative MRI techniques using multimodal comparisons
Source: PLoS One. 2025 Jul 24;20(7):e0327828. doi: 10.1371/journal.pone.0327828 (PMC12289042; doi:10.1371/journal.pone.0327828)
Supplement: S1 Table — Significant Bonferroni corrected p-values are bolded. The mean of the absolute value of the t-statistics and difference between the means of the r distributions for each pair of metric-metric correlations is also reported as an estimate of the magnitude of the difference between tissue types. (DOCX) [file pone.0327828.s008.docx]

|  | **Cortical vs WM** | | | **Subortical vs WM** | | | **Cortical vs Subcortical** | | |
| --- | --- | --- | --- | --- | --- | --- | --- | --- | --- |
|  | **t-values** | **p-values** | **Difference in mean r (cortical - WM)** | **t-values** | **p-values** | **Difference in mean r (subcortical - WM)** | **t-values** | **p-values** | **Difference in mean r (subcortical - cortical)** |
| **MTSat/FA** | 13.23 | **4.83E-22** | 0.078726333 | 23.15 | **2.66E-40** | 0.119874546 | 6.77 | **3.95E-08** | 0.041148214 |
| **PD/FA** | 3.65 | **1.90E-02** | 0.017281851 | 17.90 | **2.71E-31** | 0.125908494 | 14.96 | **1.34E-25** | 0.108626643 |
| **R1/FA** | 8.22 | **3.35E-11** | 0.048922709 | -14.74 | **3.62E-25** | -0.137660177 | -17.30 | **3.58E-30** | -0.186582885 |
| **R2/FA** | -22.88 | **7.17E-40** | -0.126249025 | -31.12 | **1.37E-51** | -0.273501634 | -16.54 | **1.02E-28** | -0.147252609 |
| **MTSat/MD** | -34.50 | **1.08E-55** | -0.219146847 | -14.43 | **1.58E-24** | -0.098312409 | 19.16 | **1.38E-33** | 0.120834439 |
| **PD/MD** | 29.74 | **8.25E-50** | 0.152598296 | 6.76 | **4.20E-08** | 0.034494929 | -21.37 | **2.07E-37** | -0.118103367 |
| **R1/MD** | -36.62 | **4.28E-58** | -0.214197213 | -32.20 | **6.14E-53** | -0.201630834 | 2.28 | 1 | 0.012566379 |
| **R2/MD** | -1.09 | 1 | -0.006810562 | -15.97 | **1.30E-27** | -0.115762127 | -16.05 | **9.07E-28** | -0.108951565 |
| **PD/MTSat** | 15.19 | **4.57E-26** | 0.126659356 | 5.19 | **5.04E-05** | 0.042923204 | -9.07 | **4.81E-13** | -0.083736153 |
| **R1/MTSat** | 6.74 | **4.72E-08** | 0.040386155 | -0.81 | 1 | -0.007669348 | -4.41 | **1.18E-03** | -0.048055503 |
| **R2/MTSat** | 9.40 | **9.15E-14** | 0.061072655 | -3.49 | **3.27E-02** | -0.029962607 | -10.28 | **1.07E-15** | -0.091035262 |
| **R1/PD** | 9.98 | **4.92E-15** | 0.08701875 | -3.93 | **6.93E-03** | -0.024709528 | -15.61 | **6.60E-27** | -0.111728277 |
| **R2/PD** | 17.70 | **6.29E-31** | 0.158328478 | 1.83 | 1 | 0.020971854 | -15.68 | **4.91E-27** | -0.137356624 |
| **R2/R1** | 4.50 | **8.34E-04** | 0.031738903 | 17.82 | **3.72E-31** | 0.159969446 | 14.24 | **3.79E-24** | 0.128230543 |
| **Absolute mean** | 14.23 |  | 0.091276 | 12.62 |  | 0.092890 | 12.25 |  | 0.096281 |
