# Supplementary material for: Assessing quantitative MRI techniques using multimodal comparisons
Source: PLoS One. 2025 Jul 24;20(7):e0327828. doi: 10.1371/journal.pone.0327828 (PMC12289042; doi:10.1371/journal.pone.0327828)
Supplement: Attachment — Submitted filename: Reply_to_Reviewers.docx [file pone.0327828.s010.docx]

Reviewer #1: The authors conducted quantitative multi-parametric and diffusion MRI in a reasonable sample size of 101 young healthy individuals (range 18-34 years). They report linear relationships between the metrics derived from these acquisitions in three tissue types (white matter, cortical grey matter, and subcortical grey matter). They then applied principal component analysis across the cortical voxels to derive latent variables and describe the cortical gradients as uncovered by the combinations of metrics loading onto the principal coordinates.

Overall, the manuscript is clearly written and is interesting. The results are interesting for researchers working with multi-modal imaging data and particularly qMRI.

General suggestions

1. The authors study metric-metric correlations in three tissues. Yet, the PCA was only conducted in the cortex. I suggest the authors replicate this to the other tissues (white matter and deep grey matter). This will be important given that some previous studies have investigated this in major white matter tracts, although with a different set of MRI parameters (e.g. combining diffusion data with ihMT and mcDESPOT by Geeraert et al 2020 https://doi.org/10.1371/journal.pone.0233244). It would be important to discuss if the resulting latent variables in white matter have similar interpretation to those in grey matter, or how similar are the latent variable interpretations in white matter derived using qMRI metrics compared to previous works. The authors state that the cortical PC6 was the most novel as it least resembled individual metrics – would all 6 PCs be informative in other tissues too? Is the more/less redundancies of the metrics in the different tissues? Do the components follow any known patterns of development or degeneration in the different tissues?

- Thank you for this excellent comment that contains some of the rationale for additional exciting approaches that we are currently working on. We are also very interested in white matter microstructure and recently released a paper that tackles multivariate white matter in a more specific way than we were able to do in the current manuscript. This work (Tremblay et al., 2024) used a specific white matter registration approach (FOD-based registrations) to ensure that white matter alignment was optimized across individuals. We had a more comprehensive set of WM metrics (DTI, NODDI, CSD, T1w/T2w) and feel that this is a much stronger basis for comparison within white matter. As a result, our group is currently exploring analyses similar to those that the reviewer has described here in the dataset described in the Tremblay paper. Similar to the paper of Geeraert et al. (2020), we are also looking to see how latent components are related to behavioural differences across a large number of individuals. A robust investigation of the important questions that the reviewer has raised here is unfortunately beyond the scope of the current manuscript. However, we also agree that they are extremely important and are in the process of addressing them with a more comprehensive dataset combining additional microstructural measurements and comprehensively characterized multi-domain behavioural data in a large sample of individuals (>700). Additionally, the main focus for the current work was to provide a comprehensive description of the metrics’ linear relationships and, as a result, in the current manuscript we have therefore chosen not to expand on the PCA analysis in the WM and subcortical GM. However, based on this comment (and others) we have now included the full comparisons between WM, cortical GM, and subcortical GM and included an additional analysis to provide an initial assessment for how similar the microsctructural profiles in each tissue type are. Please refer to our reply to Point 2 in the Discussion section of the current reviewer’s comments below for the details about these additions.
- To provide additional context on our thinking and as a result of the reviewers’ interest in this topic, we include here a short description of our approach along with some exemplary references. We proposed the Mahalanobis distance as an approach to characterize microstructural differences between individuals and/or groups while accounting for the intrinsic covariance between metrics. This approach provides a single value at each voxel (the Mahalanobis distance) that represents the overall multivariate microstructural deviation from a reference. It allows the inclusion of as many metrics as are available to the researcher without penalty and has shown excellent promise in identifying microstructural differences between groups (see example references included below). In cases where we (and other researchers) are not specifically interested in the metric loadings (i.e., the PC loadings for each individual) the Mahalanobis distance is a simple way to identify microstructural deviation.

Tremblay SA, Alasmar Z, Pirhadi A, Carbonell F, Iturria-Medina Y, Gauthier CJ, Steele CJ. 2024. MVComp toolbox: MultiVariate Comparisons of brain MRI features accounting for common information across measures. Aperture Neuro. 4.

Owen TW, de Tisi J, Vos SB, Winston GP, Duncan JS, Wang Y, et al. Multivariate white matter alterations are associated with epilepsy duration. European Journal of Neuroscience 2021;53:2788–803. https://doi.org/10.1111/ejn.15055.

Gyebnár G, Klimaj Z, Entz L, Fabó D, Rudas G, Barsi P, et al. Personalized microstructural evaluation using a Mahalanobis-distance based outlier detection strategy on epilepsy patients’ DTI data – Theory, simulations and example cases. PLOS ONE 2019;14:e0222720. https://doi.org/10.1371/journal.pone.0222720.

Guerrero-Gonzalez JM, Yeske B, Kirk GR, Bell MJ, Ferrazzano PA, Alexander AL. Mahalanobis distance tractometry (MaD-Tract) – a framework for personalized white matter anomaly detection applied to TBI. NeuroImage 2022;260:119475. https://doi.org/10.1016/j.neuroimage.2022.119475.

2. The diffusion data has potentially been under-utilized. There are several other models that can be derived from single shell data (e.g. https://www.sciencedirect.com/science/article/pii/S1053811922002956?via%3Dihub), which can add additional information. How do you expect the inclusion of more models to vary the PCA components? In addition, what is the rationale for excluding RD as it has shown some sensitivity to myelin-related pathologies (see also Blesa et al 2022 (https://doi.org/10.1162/imag_a_00017) demonstrating high correlations between RD and MTsat in white matter). In addition, do the authors think the acquisition of another shell may benefit the experiment?

- This point is well taken. While we agree that there are additional WM metrics that could be included, we also recognize that their inclusion would significantly increase the number of multiple comparisons calculated. To avoid p-hacking, we chose our dMRI metrics a priori from the diffusion tensor (FA and MD) to represent the most commonly used metrics in human MRI studies that utilize the diffusion tensor to analyze dMRI. While not exhaustive, our results with FA and MD will be informative for other researchers using, or planning to use, qMRI in their work in both GM and WM.
- For whether or not the PCA results would change with additional metrics, we expect that adding additional metrics may shift relationships somewhat, but that the general pattern would remain similar when the approach is used in healthy adults. Beyond questions in the healthy adult population, this is perhaps more interesting to consider in the context of disease and/or development – as we may expect a shift in the correlations between metrics as a result of a differential effect of pathology/development on specific biophysical tissue parameters (e.g., myelin, cell density). In this case, it may be more beneficial to include as many metrics as possible to maximize the chance that one of them will reflect pathophysiological (or developmental) change and therefore result in a differential weighting on the PCs. With this type of data, we would therefore hypothesize that adding an additional shell to the dMRI acquisition would allow additional metrics to be calculated, that may be more sensitive to the expected change. As detailed in our previous response above, while exploring all of these questions is beyond the scope of the current manuscript, we have begun to tackle them with a complementary approach in some of our other recent work (Tremblay et al., 2024).

Abstract

1. In the sentence starting with “To support the development ...” please add “and dMRI” metrics. This is because dMRI metrics can’t strictly be classified under qMRI.

- This has been clarified in the text.
  - *To support the development and common use of qMRI for cognitive neuroscience, we analysed a set of qMRI and dMRI metrics (R1, R2*, Magnetization Transfer saturation, Proton Density saturation, Fractional Anisotropy, Mean Diffusivity) in 101 healthy young adults.*

Introduction

Overall enough background information is included in the Introduction section. However, some key references seem to be missing. Some specific comments and suggestions are as follows:

1. Please define all abbreviations at first use, e.g. WM, CSF, MTR.'

- Thank you for pointing this out. We have now defined all abbreviations at first use.

2. The first sentence of the third paragraph of the Introduction needs to be clarified. The part before the brackets is confusing and contrasting with that included in the brackets. Perhaps paraphrase the entire sentence to: "One major issue is that the same MR signals can be the result of many different molecular arrangements and their concentrations"

- This sentence has been clarified. We have changed the wording as follows:
  - *One major issue is that the MR signal from a single voxel is potentially the result of many different molecular arrangements and concentrations, and therefore provides ambiguous information about the microstructural composition in that voxel* [*[Draganski et al., 2011; Tardif et al., 2016; Tardif et al., 2017]*](https://www.zotero.org/google-docs/?p2diAE)*.*

3. In the third paragraph, when discussing how latent variables can be derived from MRI metrics, the authors should also reference Chamberland et al 2019 https://doi.org/10.1016/j.neuroimage.2019.06.020.

- Thank you for this suggestion. We have integrated this into the text.
  - *Extracted latent variables can be related to microstructural features and ground-truth molecular concentrations to determine how well they map to specific tissue properties, such as disentangling microstructural differences related to diffusion hinderance from those related to orientation and fiber organization* [*[Borsboom et al., 2003; (Chamberland et al., 2019); Filo et al., 2019;*](https://www.zotero.org/google-docs/?5E1JlZ) *(Geeraert et al., 2020)].*

4. In the same sentence, the reference to Geeraert et al 2019 https://doi.org/10.1002/hbm.24706 seems misplaced as this paper did not derive latent variables, though they did analyse data from multiple acquisitions in relation to age and sex. Yet, in another paper by the same first author they did apply dimensionality reduction for dMRI, ihMT and mcDESPOT data and should be referenced instead: Geeraert et al 2020 https://doi.org/10.1371/journal.pone.0233244. Of note, the authors reference the 2019 paper in multiple places in the manuscript when referring to latent variables, so I suggest them to doublecheck the references.

- Thank you for catching this referencing error. We have corrected the in-text citations and bibliography to reflect this.

5. The sentence starting with “qMRI has other advantages, including ...”: it should include “diffusion MRI commonly used for assessing microstructural integrity” as it’s not only structural MRI that qMRI provides advantages to.

- Thank you for pointing this out, this has now been clarified in the text as follows:
  - *qMRI has other advantages, including higher reproducibility and comparability between acquisitions within and between participants than conventional structural MRI commonly used for assessing macroscopic morphological change, as well as diffusion MRI commonly used for assessing microstructural integrity* [*[Caan et al., 2019; Leutritz et al., 2020; Weiskopf et al., 2015]*](https://www.zotero.org/google-docs/?e9PBtw)*.*

6. In the 4th paragraph when discussing redundancies of information provided by MRI metrics, it is important to also cite Chamberland et al 2019, and De Santis et al 2014 https://doi.org/10.1016/j.neuroimage.2013.12.003

- We have responded to this point in our reply to point 7 below.

7. In the same paragraph, the sentence discussing previous work of metric-metric correlations could also include references to Chamberland et al 2019, Geeraert et al 2020, and Vaher et al 2022 https://doi.org/10.1016/j.neuroimage.2022.119169 - these papers have also explored metric-metric covariance within and along major white matter tracts (although two of these only focus on diffusion metrics, but exemplify the redundancy aspect). The authors could also add reference to Blesa et al 2022 (https://doi.org/10.1162/imag_a_00017) which investigated correlations between dMRI and MT-metrics in major WM tracts and across all WM voxels in a neonatal dataset.

- We have adapted this paragraph to include these references as well as the references mentioned in point 6. Please find the paragraph in its entirety below for direct reference. Just to note that the Blesa reference is cited by the author’s last name below (*Cábez).*
  - *While useful for acquiring multiple co-registered modalities, and often more robust in explaining variation in brain microstructure [De Santis et al., 2014], multiparametric methods likely contain redundancies in the information provided by each metric* [*[Callaghan et al., 2015b; Weiskopf et al., 2015; Weiskopf et al., 2021]*](https://www.zotero.org/google-docs/?TwfARH)*, especially given the fact that dMRI and qMRI metrics show high correlation with eachother [Cábez et al., 2023]. In fact, in certain circumstances, a subset of qMRI and dMRI metrics that show redundancies to one other metrics present in the analysis can be removed without significantly reducing the variance explained by the remaining metrics. As such, these redundancies have the potential to be exploited to determine which combination of metrics accounts for the most variance in the tissue of interest (and could therefore form a basis for a minimum useful multiparametric acquisition profile) and, importantly, can then be used to map putative microstructural similarities across the brain* [*[Weiskopf et al., 2015]*](https://www.zotero.org/google-docs/?VYz1RY)*. Similar to the way that microstructure exhibits differences across different tissues and regions, the relationships between metrics are also expected to vary. Previous work has explored metric-metric covariance relationships with ROI-based or segmented network approaches* [*[Seidlitz et al., 2018; Uddin et al., 2019]*](https://www.zotero.org/google-docs/?Z7m8gZ) *such as analyzing metric covariance in predetermined WM tracts of interest [Chamberland et al., 2019; Geeraert et al., 2020; Vaher et al., 2022], and a within-ROI binning approach* [*[Filo et al., 2019]*](https://www.zotero.org/google-docs/?VSSJhD)*, which rely on a priori regional delineations and averaging that may obscure subtle variability within regions. A voxel-wise approach could provide a more nuanced and comprehensive description of these relationships and help to determine if and/or how multiparametric combinations can provide additional information not found in individual metrics* [*[Paquola et al., 2019]*](https://www.zotero.org/google-docs/?EHB4S9)*.*

Methods

Overall, the methods of this paper seem robust. To reduce partial volume effects, the authors apply two different tissue segmentation toolkits and limit their analyses for voxels included in the intersection of the methods. Other methods for cortical surface generation and group co-registration follow previously published methods. Some points for clarification:

1. Please specify which methods were used for susceptibility correction and image registration were used. Please also include references.

- Thank you for pointing out this omission. The susceptibility correction and the image registration were both conducted using tools in the FSL toolkit. Specifically, the registration was conducted using FSL FLIRT, and susceptibility correction was conducted using TopUp. We have integrated this information into the main text of the manuscript within the Methods.
  - *The dMRI images were corrected for motion, eddy currents, and susceptibility distortions using the FSL TopUp toolkit [Andersson and Sotiropoulos, 2016], and the fractional anisotropy (FA), and mean diffusivity (MD) were computed from the diffusion tensor images using the FSL tools [Jenkinson et al., 2012]. Registration from native diffusion to higher resolution MPM space was conducted using FSL FLIRT and was accomplished by rigidly co-registering the b0 and R1 images within each individual, and applying the transform to FA and MD maps to bring them into R1 space (trilinear interpolation).*

2. Section 2.2 last paragraph: the authors state that magnetisation transfer maps were estimated from MPMs. Please be more specific which exact MT metric was derived: was it magnetisation transfer ratio (MTR) or magnetisation transfer saturation (MTsat). Both seem to be possible to be derived from the data at hand. Arguably, MTsat would be a better measure given that it is inherently corrects for B1+ inhomogeneities and T1 relaxation to a substantial degree.

- Apologies for the confusion, we have corrected the text to reflect that this is in fact MTsat. Thank you for catching this.

1

3. Section 2.3.1 segmentation: the authors use seemingly arbitrary thresholds for excluding voxels. Please provide references and/or reasoning for the use of these thresholds.

- Thresholds were chosen to remove/reduce partial voluming effects and to eliminate artifactual R2* signal (primarily in the temporal lobes). As there is no ground truth or existing publication with this data that has identified thresholds previously, we took a rigorous quality assurance approach to select the thresholds that we have reported in the manuscript. We have now added additional text to this section to further clarify this and contextualise the approach as follows:
  - *Cortical, subcortical, and WM segmentations for each participant were performed with standard GM segmentation in SPM12 on the R1 image* [*[Ashburner and Friston, 2005]*](https://www.zotero.org/google-docs/?WsFxR1) *and the Nighres implementation of Multiple object Geometric Deformable Model (MGDM) segmentation using FA, R1, MT, and PD* [*[Bazin et al., 2014; Bogovic et al., 2013]*](https://www.zotero.org/google-docs/?GKNKdQ)*. We utilized the multiple segmentations in combination with rigorously identified threshold cutoff values to restrict our analyses to only the tissues of interest (cortex, subcortex, WM) while excluding voxels that included partial voluming (e.g., GM that includes WM, CSF, or dura) or artifactual signal. Final masks were inspected for each individual by FC to ensure that they corresponded only with the specific tissue type (cortex, subcortex, WM). MGDM allowed for the segmentation of the cerebral cortical and subcortical GM while preserving topology, and SPM segmentations were ~~then~~ combined as follows to ensure final segmentations that reduced tissue partial voluming. WM masks were defined as the intersection between SPM WM probability maps thresholded at 0.5 and the MGDM cerebral WM. The MGDM provides a topologically correct tissue segmentation that is generally more conservative than SPM’s WM segmentation and their intersection ensured that any partial voluming effects from either approach would not be included in the final mask. GM masks were defined as the intersection between the SPM GM (probability maps thresholded at 0.95) and the MGDM labels for cerebral GM. We used a more conservative threshold for the SPM GM probability maps in this step to eliminate non-brain tissue at the pial surface to allow for a more accurate cortical extraction (see 2.3.2). As for cortical grey matter, s~~S~~ubcortical GM masks were defined as the intersection between the MGDM subcortical labels and the SPM GM thresholded at 0.95. The resulting masks retained artifactual R2* signal (mainly in the medial temporal lobe) which we removed by excluding voxels containing R2* values above 25 s^-1^ in GM (WM was not thresholded). We also used an MD threshold of 0.001 mm^2^s^-1^ x 10^-6^ to reduce any partial voluming with CSF in the GM masks. These threshold values were chosen with reference to the anatomical images and segmented tissue masks in each individual and the same values used across the entire group to ensure comparable tissue type identification. All masking and metric thresholds were chosen to restrict our analyses as closely as possible to tissue type (GM, WM) and optimally reduce partial voluming and signal artifacts. Subsequent analyses were performed on voxel-wise values extracted from the cortical sheet, WM, and subcortical GM in both hemispheres. We also repeated these analyses without any metric thresholds and found very similar results with identical significant effects (data not shown). A group surface co-registration step for both hemispheres was additionally used to project cortical results onto a common group surface for visualization as detailed below.*

4. Section 2.5.1 statistical analysis: I think the regression models to evaluate metric-metric correlations should include adjustment for repeated measures within participant as each participant contributed to the analysis with values from thousands of voxels.

- Thank you for your comment regarding the statistical analysis. While we understand the concern for correcting for repeated measurements, we do not believe that it applies in the current case and are not sure how it could potentially be applied without multiple timepoints in our data. In this analysis each individual contributes a single set of measurements for each metric (for each tissue type) and the overall density plot is the result of the concatenation of all participants’ data (for the two metrics in the given tissue type). Each participant’s contribution is unique (as they come from independent individuals) and not repeated across, for example, multiple timepoints. The resulting density plot and correlation is across the entire group and represents the overall relationships between each pair of qMRI metrics. As far as we can determine, this would not be considered the same as repeated measurements within the same individual and we therefore did not apply a correction within participants. We applied Bonferroni correction for the metric-metric correlation comparisons to correct for the repeated t-tests.

Results

1. Section 3.1 and Figure 1 legend should include a cross-reference to Supplementary figure 1.

- This has been updated in the text and in the figure caption.
  - *(c) Using data from the cortical ribbon, we plotted the median R1 values across all participants to the group template. Other metric plots can be found in supplemental figure 1.*
  - *As expected, R1 cortical maps exhibited a gradient with peaks in primary sensory and motor regions (Figure 1), a pattern that is in correspondence with what has been shown in previous literature* [*[Marques et al., 2017; Shams et al., 2019]*](https://www.zotero.org/google-docs/?acoF1U)*. Surface plots for all six metrics can be found in supplemental figure 1.*

2. Please include the figure with all PCA gradients in the main text (i.e. combine Fig 4 and supplementary fig 2) and include discussion about it in the Results and Discussion section.

- The information originally included in supplementary figure 2 has now been integrated into figure 4, and supplementary figure 2 has been removed. Consequently, supplementary figure 3 is now supplementary figure 2, and supplementary figure 4 is now supplementary figure 3. We have also expanded the figure caption for figure 4 to reflect the new information, expanded on the results shown in this figure, and included it in the discussion.
  - *PC1’s cortical gradient showed high correspondence with primary sensory and motor regions, similar to the R1 cortical map (Figure 1), as did the gradient of PC5, albeit to a lesser degree. Similarly, PC2 resembled an inverse mapping of R2* and FA gradients, while PC3 resembled PD. The most novel gradient was in the component that explained the least amount of variance in contrasts (PC6, 3.1%) (Figure 4). This gradient least resembled individual quantitative metrics and showed a structural pattern having a degree of resemblance with the Default Mode Network, with high values in the posterior cingulate cortex, medial PFC, and posterior parietal cortex* [*[Huntenburg et al., 2017; Raichle, 2015]*](https://www.zotero.org/google-docs/?fnEINV)*.*

- *Figure 4: Results of the PCA. (a) radar plots showing the weighting of each component (ranging from -1 to 1) on each PC. In brackets is the percent variance accounted for by individual PCs. Below the radar plots, each PC has been mapped back onto the brain, showing the brain regions in which the variance was most explained by the each PC. Of particular note are PC1, showing primary sensory and motor regions and PC6. Although PC6 only accounts for 3.1% of the variance in the data, this component shows an interesting cortical gradient. (b) A plot of variance accounted for in each PC*

Discussion

1. The last sentence of the first sentence should clarify that these results only apply to PCA conducted in the cortex.

- This has been clarified in the text as follows:
  - *Furthermore, we found that applying PCA to cortical gray matter data resulted in the extraction of latent space components not obvious in individual unimodal metrics in the component accounting for the least variance.*

fi

2. Given that cortical and subcortical metric-metric correlations also differed, it is important to include a further discussion about all pairwise comparisons of the correlations between the three tissues. For example, are the correlations in WM more similar to those observed in subcortical GM? Or were cortical and subcortical GM correlations more similar to one another than compared to WM? Another angle to answer these questions would be to include the PCA in the three tissues, and compare and contrast the resulting radar plots.

- Thank you for this excellent suggestion. As suggested here we have now directly compared the metric-metric pearson’s correlations between tissue types to address this point. (Please also refer to our reply to the first comment of Reviewer 1 for additional rationale for why we did not feel that PCA was the most appropriate choice here). We have now included two supplementary figures showing the pairwise correlations of WM and cortical GM, as well as WM and subcortical GM. Additionally, we have conducted an additional set of statistical analyses (described in section 2.5.2 of the methods, text included below) and included these results in section 3.2.3 and supplementary table 1, and added a paragraph to the discussion.
  - *2.5.2 Metric-metric correlation tissue type comparisons*

*We sought to identify potential differences between tissue types on their metric-metric relationships as an initial exploration for how similar these relationships are across tissues. We computed the metric-metric correlations for each individual and tissue type and then used paired t-tests to compare correlation values between each tissue type. For example, for FA vs MD each participant’s FA-MD in WM and FA-MD in cortical GM correlation r-values were entered into a paired t-test to compare this relationship between WM and cortical GM. All comparisons were Bonferroni corrected for multiple comparisons.*

And in the results:

- *3.2.3 Metric-metric correlation tissue type comparisons*

*Overall, the pairwise correlations were most similar when comparing cortical and subcortical GM, and most different when comparing cortical GM with WM. As a summary estimate of the difference between metric-metric relationships across tissue types, the mean absolute value of the t-values across all comparisons was 14.23 for cortical vs WM, 12.62 for subcortical vs. WM, and 12.25 for cortical vs. subcortical. Across all comparisons, there were only a few relationships that were not significantly different after multiple comparisons correction. Cortical vs WM: R2/MD (t= -1.09, p=1). Subcortical vs WM: R1/MTsat (t= -0.81, p=1), R2/PD (t=1.83, p=1). Cortical vs subcortical: R1/MD (t=2.28, p=1).*

Additionally, we have included these results in the discussion as follows:

- *Our results when comparing WM with cortical and subcortical GM separately provide additional complementary evidence for the similarity and differences between tissue types. We found that the correlations between metrics in subcortical GM resembled more closely those found in WM than those found in cortical GM (mean t in cortical vs WM = 14.23, mean t in subcortical vs WM = 12.62). However, while this provides a holistic comparison of the metric-metric correlations across tissue types, correlations may vary across different regions within each tissue. For example, regional cortical differences in cyto- and myelo-architecture or tract-specific differences in myelination or density would be expected to alter the relationships that we have identified here. Future work could explore how metric-metric correlations spatially vary across tissues in healthy control populations as a normative comparison to identify more subtle region-specific changes that may serve as early biomarkers for disease. Complementary approaches (e.g., in white matter [Chamberland et al., 2021; Tremblay et al, 2024]) could extend this work to define spatially varying patterns of multivariate similarity in metric-metric correlations in specific tissues or regions in individual participants.*

Lastly, please find below all new figures relating to this additional statistical analysis:

Supplementary Figure 5: Distribution of R^2^ values across participants for all pairwise correlations (cortical GM in green, WM in red). This shows that the relationships between metrics were highly consistent across individuals.

Supplementary Figure 5: Distribution of R^2^ values across participants for all pairwise correlations (WM in red, subcortical GM in indigo). This shows that the relationships between metrics were highly consistent across individuals.

Supplementary Figure 6: Pairwise correlations in WM (above the diagonal) and subcortical GM (below the diagonal) for all participants’ data. P-values for all pairwise correlations were very small (<0.001), however were not stated explicitly as the small values of p do not necessarily relate to higher explained variance. R^2^ values for each pairwise correlation are shown above their respective graphs Data along the diagonal shows the histograms for each metric in subcortical (purple) and WM (red). Metric thresholds were implemented for R2^*^ and MD to reduce partial volume effects and artifacts, and are visible as sharp cutoffs in the figure (see Method). Pairwise density plots are depicted in log space to show the full range of data.

Supplementary Figure 7: Pairwise correlations in WM (above the diagonal) and cortical GM (below the diagonal) for all participants’ data. P-values for all pairwise correlations were very small (<0.001), however were not stated explicitly as the small values of p do not necessarily relate to higher explained variance. R^2^ values for each pairwise correlation are shown above their respective graphs Data along the diagonal shows the histograms for each metric in cortical (green) and WM (red). Metric thresholds were implemented for R2^*^ and MD to reduce partial volume effects and artifacts, and are visible as sharp cutoffs in the figure (see Method). Pairwise density plots are depicted in log space to show the full range of data.

3. How can these findings of MRI metric covariance be extrapolated to other species or developmental time points?

- This is an interesting addition, thank you for allowing us to elaborate on it. This analysis would certainly be very interesting to conduct over developmental time points, in other species, or in patients with other pathologies. Given that there are current datasets (e.g., ABCD) that longitudinally follow individuals over development, this could be a very interesting set of analyses to undertake (we invite the reviewer to contact us to potentially discuss this further, if there is interest!). We would expect that different developmental timepoints would have differential weighting on dendritic arborization and pruning, which would make this addition especially interesting. We would hypothesize that there would be relative changes in the relationships between metrics more tied to axons and the other metrics (e.g., FA, MT, R1, vs others), differentially across regions over the course of development. For example, in the frontal lobes and WM of the superior longitudinal fasciculus from puberty to young-adult. While we think it too speculative to go into this point in great detail (given that we do not have this type of data in the current manuscript), we have added additional text to highlight and expand on these ideas in two locations within the discussion, as copied below.
  - *The normative R1-MT relationships described here could potentially be affected by pathological conditions that affect macromolecular proportions, such as Alzheimer’s tau pathologies and white matter hyperintensities, and baseline relationships would then be useful for research in these domains* [*[van Es et al., 2006; Hentschel et al., 2004; Wardlaw et al., 2016]*](https://www.zotero.org/google-docs/?VbI0Eu)*. Repeating this analysis on a sample with various neurodegenerative diseases would increase the generalizability of results, and perhaps give us insight on how these diseases affect the brain if the principal components that drive the variance show differential patterns across pathologies.*
  - *Past research has found that R1 and diffusion have differential variation throughout the lifespan, further supporting a difference in the information captured by these metrics, and suggesting that varying R1-dMRI correlations may be informative about health and ageing related issues* [*[Yeatman et al., 2014]*](https://www.zotero.org/google-docs/?KhXYfq)*. We would also expect that the age-related changes in dMRI metrics would be region-specific, as aging affects myelination differentially across regions. It would ther be beneficial to perform a similar analysis across developmental timepoints in addition to the present single-timepoint whole brain analysis.*

4. When discussing the correlations between FA and R1 it would be good to include a reference to Blesa et al 2022 using a neonatal dataset as it found something similar across all WM voxels (low correlations between FA and MTsat), while the correlations were different withing major tracts (FA correlations with MTsat stronger).

- *Thank you for pointing this out, we have now included this reference in this section of the discussion.*
  - *... to more accurately specify this relationship. In addition, correlations with FA have been found to be stronger when restricted to anatomically defined tracts rather than across all white matter [Cábez et al., 2023]. Our findings are in line with...*

Reviewer #2: The authors report the correlation between standard qMRI parameters and selected DTI metrics (FA and MD) in grey matter and white matter regions. Data was collected on 101 young subjects with relatively small age variation hence minor age effects were introduced in the analysis. GM was divided into sub-cortical GM and cortical GM (voxels were extracted along a skeleton of cortical GM). Correlation results were as expected from the literature. The authors explored the usage of principal component analysis (PCA) of the parameters from qMRI and DTI and mapped the components back to the brain to see if they can explain anatomical relations. I believe it is a nice data set collected, and it is informative to inspect the correlations, but the scientific contribution is not clear to me. The discussion reads as a confirmation of the many existing studies and does not add a new dimension. The PCA seems interesting, but the authors did not take it further and it is a kind of hanging e.g. relating it to cortical regional (from atlas) and/or tract-based atlases allowing the drawing of some functional-related conclusions.

- We thank the reviewer for the helpful comments and thoughtful suggestions for improving the manuscript. We are in agreement that our dataset is interesting and that this work sheds light on qMRI by inspecting the correlations between quantitative imaging metrics. As the reviewer notes, our results are in very good alignment with the literature and, further, we have presented a more comprehensive description of the relationships of six qMRI metrics within white and grey matter, all within a single dataset. As a result of the Reviewers’ helpful comments, the presented relationships in the revised version of the manuscript are complete across tissue types and compare WM-cortical ; WM-subcortical ; and cortical-subcortical. The impact/usability of our results have been further enhanced by incorporating the Reviewers’ suggested additions.
- We also agree that the PCA is an interesting addition. However, as detailed in our reply to the first and second comments of Reviewer 1, the inclusion of additional exploration of the PCA is beyond the scope of the current manuscript. We have ongoing work in a set of participants from the HCP that is exploring the latent component space in a complementary way (using the Mahalanobis distance, as described in the first response to Reviewer 1). We also agree that it will be important for the field to identify regional cortical and tract-specific relationships that can then be related to individual differences in behaviour (or across development, disease states). We believe that this is best done in a large population dataset (such as the HCP or UK Biobank) where a comprehensive set of behavioural measurements has also been acquired – thus allowing for a comprehensive decomposition of the multivariate links between multiple qMRI measurements of tissue microstructure and behaviour.

Section 2.5.2. please make clear what metrics refer to. The authors should elaborate on the PCA method part.

- Thank you for pointing this out. To address your comment we have added additional text to help clarify and included explicit reference to all of the metrics that were included in the PCA in parentheses. Please note that this is now section 2.5.3 in the revised manuscript.
  - *As an additional exploratory step, we performed model-free dimensionality reduction with Principal Component Analysis (PCA) in cortical tissue to visualize the latent variables that drive the variance within the data. All six metrics (R1, R2*, MT, PD, FA, and MD) in each of these tissues from all participants were z-scored and used as input variables to a PCA in scikit learn* [*(Pedregosa et al., 2011)*](https://www.zotero.org/google-docs/?iENvqo) *in Python (3.7), and all six PCs were assessed. Each participant’s data was then transformed into the PCs’ subspace for visualization, and transformed into the common group space. This allowed us to compute the median across participants and visualize our results on the cortical surface template.*

Figure 1: Please add arrows for the reader to follow the explanation, especially the cortex part. The figure text also needs to be elaborated.

- Thank you for this suggestion. We have tried to further clarify the figure caption to better explain the cortical mapping of R1 values. We have explicitly pointed out the locations of high gradient values in the caption and so have not added arrows to the figure. If there are other specific parts of the images that the reviewer thought could be better highlighted, we are open to additional suggestions on how to clarify the image (and caption).
  - *Figure 1: Overview of segmentation results. (a) The six metrics used were captured in 101 participants. (b) A sample brain with a red mask of the middle cortical ribbon as a semi-transparent overlay on R1. (c) Using data from the cortical ribbon, we plotted the median R1 values across all participants to the group template. The gradient here corresponds to the R1 values mapped back onto the brain, and shows overall higher values in the sensory and motor areas of the cortex that decrease with distance. Other metric plots can be found in the Supplementary Figure 1.*

P18. “… showing little to no partial voluming …” in relation to what?

- Thank you for this feedback. As this was unclear, we have elected to remove this phrase from the figure caption.

Section 3.4. The authors refer several times in the text to a “gradient” but it is unclear what this refers to.

- Our apologies for the confusion. The term gradients here refers to the spatially varying pattern of the derived values of the principal components. They are a direct comparison to the similar gradients shown of single metrics, for example R1 and PC1 show similar gradient patterns, as R1 was highly weighted in PC1. We have clarified the first sentence of section 3.4 as follows:
  - *The results of the PCA revealed various patterns of cortical gradients, i.e. the derived values of the principal components mapped back onto the surface of the cortex for visualization.*

P18 “…excellent …” What do the authors mean by “excellent”?

- Thank you for pointing this out. We agree that the word “excellent” without any point of reference is unnecessary, and so we have removed it.

Section 3.2 and figure 2. The authors state that all metrics are significantly correlated but when inspecting the correlation plot in Figure 2 is not convincingly supporting the result. E.g. MT vs FA and PD vs PD etc. I suggest showing the correlation in each plot of Figures 2 and 3 to support the statistics.

- As noted, all correlations were statistically significant; however, the confusion may have arisen because the density plots are displayed in log-space. As in other similar work (e.g., Cábez et al, 2023) , this was required to enable us to show the large range of densities across the plot. Above each figure we include the proportion of variance accounted for (r-squared) value and the distribution of these values across individuals in the Supplementary Figures (2-5) and, in addition, we have made this explicit in each figure legend. At the suggestion of the first Reviewer, we have also added Supplementary Figures 6-7 to show the complete relationships between all pairs of tissue types (WM, cortical GM, subcortical GM).
- We have added the following in all figure captions for these plots to clarify.
  - P-values for all pairwise correlations were very small (<0.001), however were not stated explicitly as the small values of p do not necessarily relate to higher explained variance. R^2^ values for each pairwise correlation are shown above their respective graphs.

Cábez, M. B., Vaher, K., York, E. N., Galdi, P., Sullivan, G., Stoye, D. Q., Hall, J., Corrigan, A. E., Quigley, A. J., Waldman, A. D., Bastin, M. E., Thrippleton, M. J., & Boardman, J. P. (2023). Characterisation of the neonatal brain using myelin-sensitive magnetisation transfer imaging. Imaging Neuroscience, 1, 1–17. <https://doi.org/10.1162/imag_a_00017>

`

P21 ” …such as Alzheimer’s tau pathologies and white matter hyperintensities,…” Please elaborate.

- Thank you for this feedback. We agree that the discussion of generalizability and future directions required some elaboration. We have addressed this point in our earlier response regarding possible investigations at various developmental timepoints and across-species.

P22: “Interestingly, the R1-R2* correlation was weaker in WM where we might expect…” Please elaborate on this sentence. It is unclear to me why this is the case.

- Here we were implicitly referring to research linking both metrics to myelin and iron concentration, and we have now added additional text to elaborate on this.
  - *Interestingly, the R1-R2* correlation was weaker in WM where we might expect it to be highest based on their shared link with myeloarchitecture. Both these metrics are linked to myelin and iron concentrations [Lutti et al., 2014; Marques et al., 2017, van der Weijden et al., 2021], and so it would follow that they would have higher correlation than what we observed. One potential reason for this is that R2* is known to be affected by white matter fiber orientation relative to the main magnetic field (B0)* [*[Kor et al., 2019; de Pasquale et al., 2013]*](https://www.zotero.org/google-docs/?jAFBuK)*. This orientation-dependent effect could lead to variation in R2* that does not arise from myeloarchitecture or iron and therefore decreases the strength of the correlation within the white matter. This effect is not expected to be significant within grey matter since there is no predominant net orientation of fibers at voxel resolutions.*
